# CRISPR/Cas9-Mediated Genome Editing Reveals *Oosp* Family Genes are Dispensable for Female Fertility in Mice

**DOI:** 10.3390/cells9040821

**Published:** 2020-03-28

**Authors:** Ferheen Abbasi, Mayo Kodani, Chihiro Emori, Daiji Kiyozumi, Masashi Mori, Yoshitaka Fujihara, Masahito Ikawa

**Affiliations:** 1 Department of Experimental Genome Research, Research Institute for Microbial Diseases, Osaka University, Suita, Osaka 5650871, Japan; ferheen11abbasi@gmail.com (F.A.); kodani@biken.osaka-u.ac.jp (M.K.); emoric@biken.osaka-u.ac.jp (C.E.); kiyozumi@biken.osaka-u.ac.jp (D.K.); mmori@cdb.riken.jp (M.M.); fujihara@ncvc.go.jp (Y.F.); 2 Graduate School of Medicine, Osaka University, Suita, Osaka 5650871, Japan; 3 Graduate School of Pharmaceutical Sciences, Osaka University, Suita, Osaka 5650871, Japan; 4 Immunology Frontier Research Center, Osaka University, Suita, Osaka 5650871, Japan; 5 RIKEN Center for Biosystems Dynamics Research, Kobe, Hyogo 6500047, Japan; 6 Department of Bioscience and Genetics, National Cerebral and Cardiovascular Center, Suita, Osaka 564-8565, Japan; 7 The Institute of Medical Science, The University of Tokyo, Minato-ku, Tokyo 1088639, Japan

**Keywords:** fertility, infertility, Plac1, egg, zona-pellucida-like domain

## Abstract

There are over 200 genes that are predicted to be solely expressed in the oocyte and ovary, and thousands more that have expression patterns in the female reproductive tract. Unfortunately, many of their physiological functions, such as their roles in oogenesis or fertilization, have yet to be elucidated. Previous knockout (KO) mice studies have proven that many of the genes that were once thought to be essential for fertility are dispensable in vivo. Therefore, it is extremely important to confirm the roles of all genes before spending immense time studying them in vitro. To do this, our laboratory analyzes the functions of ovary and oocyte-enriched genes in vivo through generating CRISPR/Cas9 KO mice and examining their fertility. In this study, we have knocked out three *Oosp* family genes (*Oosp1*, *Oosp2*, and *Oosp3*) that have expression patterns linked to the female reproductive system and found that the triple KO (TKO) mutant mice generated exhibited decreased prolificacy but were not infertile; thus, these genes may potentially be dispensable for fertility. We also generated *Cd160* and *Egfl6* KO mice and found these genes are individually dispensable for female fertility. KO mice with no phenotypic data are seldom published, but we believe that this information must be shared to prevent unnecessary experimentation by other laboratories.

## 1. Introduction

According to a study conducted by the World Health Organization in 2012, 1.9% of women aged 20–44 were unable to have their first child [1]. The burden of infertility on women has remained constant from 1990 to date. Studies show that women tend to blame themselves for infertility [2], even though half of all cases of infertility are linked to their male counterparts [3]. The genetic bases of female infertility are not fully understood, but it can be linked to ovulation disorders [4], tubal infertility [5], endometriosis [6], and implantation disorders [7], among many others.

It has been estimated that 50% of infertility cases are genetic, yet few genes have been shown to cause or to be strongly associated with primary infertility [8], which affects germ cells leading to developmental arrest and cell death. Thankfully, due to assisted reproductive technologies (ART), many infertile women can successfully have children, depending on their particular cause of infertility. For example, in vitro fertilization (IVF) bypasses the necessary step of sperm migration through the fallopian tubes. A woman who has tubal infertility can have her oocytes extracted, fertilized, and directly implanted into her uterus. Surrogacy is also on the rise. Out of almost 2 million cases of ART performed in 1999–2013, almost 2% used a gestational carrier [9]. However, there are increased risks of congenital abnormalities in children born using ART [10], indicative that genetic defects may still play a role. Fully understanding female fertility is key to supporting the reproductive health of female patients and the wellbeing of their children.

Due to ethical concerns, experimentation on humans is limited, so we are restricted to animal models. Little is understood about how genes cause reproductive issues in women, which is why it is important to use animal models to uncover the genetic foundation of these conditions. While most studies on oocytes have been conducted on nonmammalian species, such as the famous *Xenopus*, we find that 99% of mouse genes are comparable to humans [11]. This striking similarity allows us to clarify the function of genes that are evolutionarily linked and can suggest phenotypic parallels in humans. By creating knockout (KO) mice, we can investigate how essential a deleted gene is by assessing their fertility. The in vivo KO approach has been used in recent years to uncover genes that are indispensable for female reproduction. For example, JUNO (also known as IZUMO1R) is a GPI-anchored protein found on the plasmalemma of mammalian oocytes that recognizes IZUMO1, its partner on spermatozoa, and is essential for fertilization [12]. JUNO-null female mice were completely infertile, unable to produce any offspring. Similarly, two genes in the zona pellucida (ZP) glycoprotein family were also shown to be indispensable for female fertility [13]. When ZP2 and ZP3 KO mice were created, the oocytes lacked a zona pellucida around the oocyte, leading to infertility. On the other hand, ZP1 KO mice showed reduced fertility [14], showing that ZP1 individually is not completely essential for fertility. Thus, to uncover and report dispensable fertility factors is just as important for basic science research as uncovering genes that are essential.

A new tool has allowed us to produce genetically modified organisms quicker than ever before. The CRISPR/Cas9 system has transformed the reproduction field due to its ability to rapidly and effortlessly knock out genes to understand their effects in vivo [15]. This system is sustainable, efficient, and low cost. While there are many modes to deliver the CRISPR/Cas9 system to fertilized oocytes, we used two main strategies in this study—pronuclear injection of a plasmid containing the target guide RNA sequence in addition to the *Cas9* gene [16] and electroporation of crRNA/tracrRNA/Cas9 ribonucleoprotein complexes into zona-intact mouse zygotes [17]. During our in vivo screening, we uncovered five ovary-enriched genes with human orthologs that are individually dispensable for female fertility in mice: *Oosp1*, *Oosp2*, *Oosp3*, *Cd160*, and *Egfl6*. In this paper, we will focus on the *Oosp* family (*Oosp1*, *2*, and *3*), which was previously identified as an ovary-specific genomic cluster with an N-terminal zona-pellucida-like domain [18,19].

## 2. Materials and Methods

### 2.1. Animals

All experiments involving animals were approved by the Institutional Animal Care and Use Committees of Research Institute for Microbial Diseases, Osaka University (Osaka, Japan) and were conducted in compliance with the guidelines and regulations for animal experimentation. All wild-type mice used in the laboratory were purchased from CLEA Japan, Inc. (Tokyo, Japan) or Japan SLC, Inc. (Shizuoka, Japan). Mutant mice used in this study are available through Riken BioResource Center, Ibaraki, Japan (http://en.brc.riken.jp/) (RBRC numbers: *Oosp1*, 05720; *Oosp2*, 10334; *Oosp3*, 10335; *Oosp* family, 10840; *Cd160*, 9844; *Egfl6*, 9961) or Center for Animal Resources and Development (CARD), Kumamoto University, Kumamoto, Japan (http://cardb.cc.kumamoto-u.ac.jp/transgenic/) (CARD ID: *Oosp1*, 2138; *Oosp2*, 2704; *Oosp3*, 2705; *Oosp* family, 2820; *Cd160*, 2464; *Egfl6*, 2516).

### 2.2. Phylogenetic Tree and Protein Multiple Sequence Alignment

The phylogenetic tree (method: UPGMA) and protein multiple sequence alignment were created by GENETYX (GENETYX, Tokyo, Japan) with the amino acid sequences. Sequences were downloaded from the Ensembl database [20].

### 2.3. RT-PCR and RNAseq Analysis

RNA was isolated and purified from multiple adult tissues with TRIzol (15596018, Thermo Fisher Scientific, Waltham, MA, USA). All tissues (brain, heart, kidney, liver, lung, spleen, thymes, testis, ovary, uterus, oviduct) were collected from C57BL/6N mice. RNA was reverse transcribed to cDNA using SuperScript III first-strand synthesis system (18080051, Thermo Fisher Scientific). PCR was performed using KOD FX Neo (KFX-201, Toyobo, Osaka, Japan). Primers used for PCR are listed in Table S1.

For RNAseq analysis, GV oocytes were collected from the ovary of 3-week-old females that had been injected with pregnant mare serum gonadotropin (PMSG) (5 units, ASKA Pharmaceutical, Tokyo, Japan). Metaphase II (MII) stage oocytes and cumulus cells were collected from the oviducts of 3-week-old females that had been injected with PMSG (5 units), followed 48 h later by human chorionic gonadotropin (hCG) (5 units, ASKA Pharmaceutical, Tokyo, Japan). Total RNAs of mouse oocytes and cumulus cells were extracted using SMART-seq v4 Ultra Low input RNA Kit for sequencing (Takara Bio USA, Inc., Mountain View, CA, USA). The resulting libraries were sequenced using HiSeq 2500 (Illumina, San Diego, CA, USA).

### 2.4. Generation of KO Mice with Conventional Approach

The mouse *Oosp1* gene targeting vector (PRPGS00059_A_F11) was retrieved from the International Knockout Mouse Consortium (IKMC) [21]. After linearization with *Asi*SI digestion, the targeting vector was electroporated into EGR-G101 ([*CAG/Acr-Egfp*]×[*CAG/Acr-Egfp*]C57BL/6NCr) embryonic stem (ES) cells, and colonies were screened as previously described [22]. To disrupt the *Oosp1* gene, exon 2 was replaced with an IRES:lacZ trapping cassette and a floxed promoter-driven neo cassette. A diphtheria-toxin-A-chain (DTA) expression cassette was used for negative selection. After G418 selection, 24 of 48 drug-resistant clones had a homologous recombination event after PCR analysis. The mutant ES cell clones were injected into eight-cell-stage ICR embryos, and the chimeric blastocysts were transferred into the uterine horns of E2.5 pseudopregnant ICR females the next day. The obtained chimeric males were mated with B6D2F1 females for germline transmission. Offspring from heterozygous intercrosses were genotyped by PCR. Both a 532 bp band as the wild-type allele and a 331 bp band as the KO allele were amplified by PCR (Table S4). PCR was performed using KOD FX Neo.

### 2.5. Generation of KO Mice with CRISPR/Cas9 System

Most of the mouse lines were generated by CRISPR/Cas9 genome editing technology. Single guide RNAs (sgRNAs) were designed using the web tool CRISPRdirect (https://crispr.dbcls.jp/) [23] and their cleavage efficiency was evaluated by transfecting HEK293T cells with pCAG-EGxxFP (Addgene #50716) plasmids as described previously [16]. Wild-type embryos were genetically modified in vitro through the CRISPR/Cas9 system. B6D2F1 females were superovulated and paired with wild-type B6D2F1 males. Zygotes that had either indel or null mutations in the genes of interest were generated by (1) microinjecting pX330 plasmids expressing sgRNAs and Cas9 into the pronuclei of zygotes [16] or (2) electroporating two-pronuclear zygotes with crRNA/tracrRNA/Cas9 ribonucleoprotein complexes using a NEPA21 Super Electroporator (Nepa Gene, Chiba, Japan) [17,24]. The eggs were incubated in KSOM media overnight, and the two-cell-stage embryos were transferred into the oviducts of pseudopregnant ICR females. The pups obtained were genotyped by PCR and confirmed by Sanger sequencing. After genotyping, the F0 mice went through serial mating to generate homozygous mutant offspring. Primers and oligonucleotides as well as PCR conditions are summarized in Table S4. PCR was performed using KOD FX Neo (KFX-201, Toyobo, Osaka, Japan).

### 2.6. Fertility Test for KO Female Mice

Sexually mature KO female mice (7–20 weeks old) and wild-type B6D2F1 mice were caged with eight-week-old wild-type male mice for at least one month. During the fertility test, the number of pups was counted at birth. The average litter size for each mouse line was calculated by dividing the total number of pups by the number of litters.

### 2.7. Ovary Collection and HE Staining

Pregnant mare serum gonadotropin (PMSG) (5 units) was injected into the abdominal cavity of females, followed by human chorionic gonadotropin (hCG) (5 units) 48 h after PMSG. Nine hours after hCG, ovaries were dissected from hormone-primed females, fixed in Bouin’s fluid (Polysciences, Inc., Warrington, PA, USA), and embedded in paraffin wax. Paraffin sections (5-µm) were stained with Mayer’s hematoxylin solution (FUJIFILM Wako Pure Chemical, Osaka, Japan) followed by counterstaining with 0.3% eosin (FUJIFILM Wako Pure Chemical, Osaka, Japan). The sections were observed with phase contrast microscopy.

### 2.8. Sperm-ZP Binding Assay and In Vitro Fertilization (IVF)

To conduct the sperm ZP-binding assay and IVF, spermatozoa were obtained from the caudal epididymis of adult B6D2F1 males and cultured in TYH medium for 2 h at 37 °C under 5% CO_2_ [25]. Oocytes at the second metaphase (MII) stage were collected as described above. The sperm-ZP binding assay was performed with cumulus-free oocytes. Cumulus cells were removed by treatment with 330 µg/mL hyaluronidase (Sigma-Aldrich, St. Louis, MO, USA) for 5 min. Spermatozoa that were incubated for 2 h were added to a drop of TYH medium containing cumulus-free oocytes at a final density of 2 × 10^5^ spermatozoa/mL. Thirty minutes after mixing the spermatozoa, oocytes were fixed with 0.25% glutaraldehyde. The sperm-bound eggs were observed with Olympus IX73 microscopy (Tokyo, Japan). To see embryonic development, spermatozoa that were incubated for 2 h were added to a drop containing cumulus-intact oocytes at a final density of 2 × 10^5^ spermatozoa/mL. Eight hours after adding the spermatozoa, two-pronuclear (2-PN) eggs were collected and moved into potassium-supplemented simplex optimized medium (KSOM) for further observation. Embryos were continuously observed for 4 days after IVF.

### 2.9. Statistics

Statistical analyses were performed using Student’s t-test (two-tailed), and only *p*-values less than 0.05 are indicated (*). Error bars are shown as standard deviation (s.d.) if not indicated otherwise.

## 3. Results

### 3.1. In Silico Experimental Strategies to Discover Ovary-Enriched Genes for Functional Analysis

**Figure 1:**
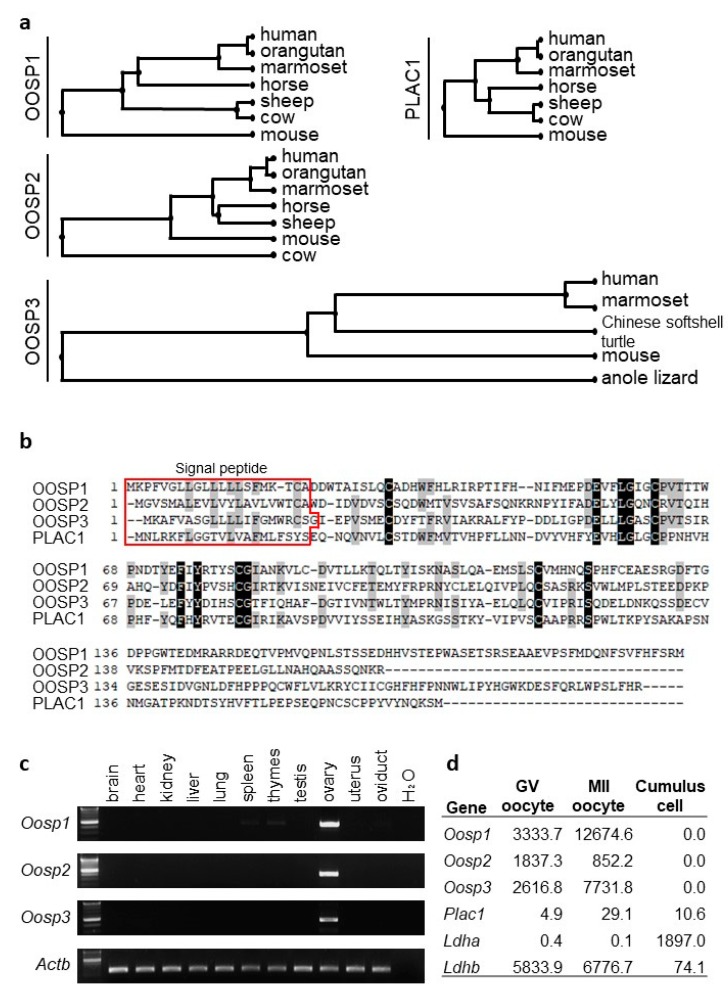
Conservation of *Oosp* family genes between species and tissue expression analysis. (**a**) Phylogenetic trees. (**b**) Amino acid homology between OOSP family. All four genes have a signal peptide sequence as coded by amino acids 1-21. (**c**) RT-PCR analysis using multiple tissues. Actin beta (*Actb*) was used as the control. (**d**) Expression levels in germinal vesicle (GV) oocytes, metaphase II (MII) oocytes, and cumulus cells. *Ldha* and *Ldhb* are shown as the control. FPKM: fragments per kilobase of exon per million reads mapped.

Using a variety of bioinformatic websites, we started by identifying potential target genes that may be important for female reproduction. There were two main goals during the search—to find genes that were enriched in the female reproductive system and had high conservation between humans and mice. We assumed that genes that are conserved between humans and mice would be essential for oocyte development. Using Unigene, we found 51 ovary-restricted and 122 oocyte-restricted genes. Once we made a list of all viable candidates, we systematically conducted RT-PCR experimentation to ensure that the gene was enriched in the female reproductive tract. The *Oosp* family (*Oosp 1, 2,* and *3*), as mentioned previously, was identified as an ovary-specific genomic cluster with an N-terminal zona-pellucida-like domain [16,17]. All three have human orthologs, as determined by Ensembl [20]. According to HGNC [26], there are six genes in the *Oosp* family: OOSP1, 2, 3, 4, 4A, 4B, and PLAC1. However, only *Oosp1, 2, 3,* and *Plac1* have mouse orthologs, and *Plac1* KO mice were fertile [27]. Further research shows that the *Oosp* family genes are conserved between a variety of mammalian species (Figure 1a). In addition, there is significant homology between the OOSP family proteins (Figure 1b). All four genes have a signal peptide, coded by amino acids 1-21. Further in silico research found that two other oocyte-enriched genes, *Cd160* and *Egfl6,* are also conserved in mammals and have human orthologs.

### 3.2. RT-PCR and FPKM Analysis Confirms Ovary-Enriched Expression in Mice

To confirm that the *Oosp* family genes were expressed in the ovary, we performed RT-PCR using cDNA obtained from adult mouse tissues with specific primers (Table S1). All three genes were enriched in the ovary (Figure 1c). Further, RNAseq analysis showed that the *Oosp* family genes were highly expressed in the GV and MII oocyte (Figure 1d). *Cd160* and *Egfl6* were also found to be oocyte-enriched by RT-PCR and RNAseq analysis (Figure S1).

### 3.3. Generation and Phenotypic Analysis of Oosp1 Mutant Mice Using Traditional KO Methods

**Figure 2:**
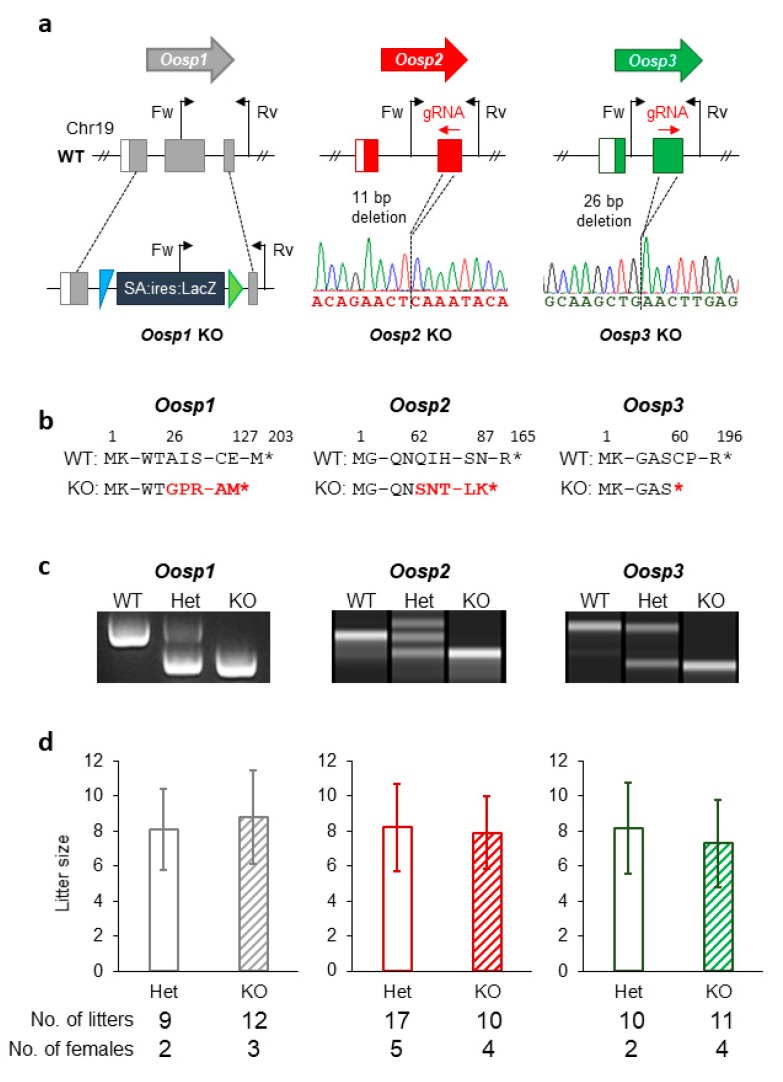
Generation of KO mice and their fertility analysis. (**a**) Genomic structure and KO strategy of mouse *Oosp1*, *Oosp2*, and *Oosp3*. Black arrows: primers used for genotyping. Fw: Forward primer; Rv: Reverse Primer. Colored boxes: coding regions. WT: wild-type. (**b**) Truncated amino acid sequence. Homologous recombination in *Oosp1* region induced IRES sequence instead of second exon and resulted in premature stop codon at 127 amino acid. The 11 bp deletion in the exon in *Oosp2*^−11/−11^ mice caused a Q62S mutation resulting in premature stop codon introduced 25 amino acids later. The 26 bp deletion in the exon in *Oosp3*^−26/−26^ mice caused mutation in C60 to stop codon. (**c**) Genotype verification of *Oosp1*, *Oosp2*, and *Oosp3* KO mice by genomic PCR. WT: wild-type. (**d**) Average litter size of control and KO females.

To investigate how essential the *Oosp* family genes are in female reproduction, we started by deleting each gene individually using traditional strategies via KOMP resources. We followed the International Knockout Mouse Consortium (IKMC) guidelines to make the *Oosp1* targeting vector [21] (Figure 2a,b). After linearization with AsiSI digestion, the vector was electroporated into embryonic stem cells and screened [22]. We replaced exon 2 with an IRES:lacZ gene-trapping cassette and a floxed promoter-driven neo cassette. After obtaining mutant ES clones, we injected them into eight-cell-stage embryos to produce chimeric male mice (Table S2). We subsequently mated these male mice with wild-type females to obtain heterozygotes and then mated the heterozygotes to produce homozygous female mice. Heterozygous mutant pairs produced 22% homozygous offspring (17 out of 54 pups in six litters), showing that homozygous mice were not embryonically lethal. Offspring were genotyped by PCR and were found to have the gene-trapping cassette successfully inserted into the gene (Figure 2c). The insertion of the gene-trapping cassette resulted in a frameshift mutation of A26G with a premature stop codon introduced 99 amino acids later (Figure 2b). *Oosp1^-/-^* mice were phenotypically analyzed alongside their wild-type or heterozygous controls. The mutant mice had no overt developmental or behavioral abnormalities. To test the fertility of female mice, adult KO females were mated with wild-type males for several months. All of the *Oosp1^-/-^* KO female mice were fertile, with an average of 8.8 ± 2.7 pups per litter (Figure 2d).

### 3.4. Generation and Phenotypic Analysis of Oosp2 and Oosp3 Mutant Mice Using the CRISPR/Cas9 System

When the CRISPR/Cas9 system was developed [28,29], we used it to produce *Oosp2* and *Oosp3* single KO mice (Figure 2a). We designed guide RNAs (gRNAs) that had the fewest predicted off-target hits using CRISPRdirect [23] and are summarized in Table S2. After confirming the activity of the gRNAs in vitro, we inserted the target gRNA sequence into the pX330 plasmid, which also contains the humanized Cas9 sequence, and microinjected 5 ng/μL of the plasmid into the pronuclei of fertilized oocytes [16]. Eighty-seven *Oosp2* and 102 *Oosp3* gRNA-injected two-cell embryos were transplanted into the oviducts of pseudopregnant female mice. Of the pups genotyped, two *Oosp2* and three *Oosp3* mutants were obtained (Table S3). Subsequent mating resulted in an *Oosp2* KO mouse with an 11 bp deletion (*Oosp2^−11/−11^*) and an *Oosp3* KO mouse with a 26 bp deletion (*Oosp3^−26/−26^*) (Figure 2a,b). These deletions were confirmed by PCR and sequencing analysis (Figure 2c). The primers and amplification conditions for each gene are summarized in Table S4. The 11 bp deletion in the *Oosp2^−11/−11^* mice resulted in a frameshift mutation of Q62S with a premature stop codon introduce 25 amino acids later (Figure 2b), while the 26 bp deletion in the *Oosp3^−26/−26^* mice resulted in a frameshift mutation of C60X with a premature stop codon (Figure 2b).

Both KO mouse lines were phenotypically analyzed alongside their wild-type or heterozygous controls. The mutant mice were viable, with no overt abnormalities. To test the fertility of female mice, adult KO females were mated with wild-type males for several months. Similar to the *Oosp1^-/-^* females, both the *Oosp2^−11/−11^* and *Oosp3^−26/−26^* KO female mice were fertile, with averages of 7.9 ± 2.1 and 7.3 ± 2.5 pups per birth, respectively (Figure 2d).

### 3.5. Generation and Phenotypic Analysis of the Oosp Family Deletion Mice

**Figure 3:**
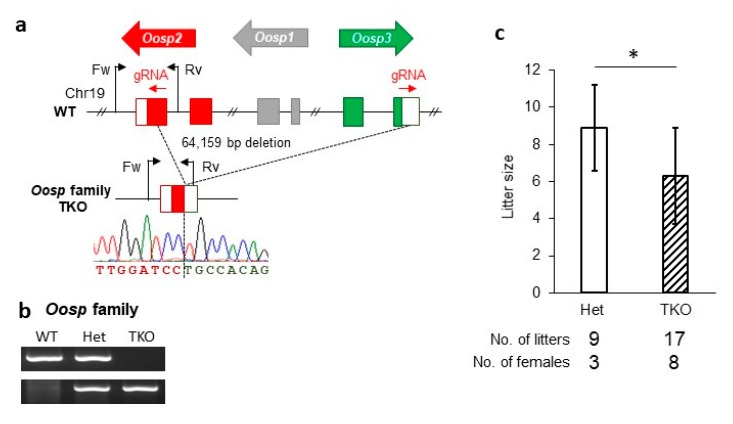
Generation of *Oosp* family KO mice and their fertility analysis. (**a**) Genomic structure and KO strategy of *Oosp* family (*Oosp1, Oosp2* and *Oosp3*). WT: wild-type. (**b**) Genotype verification of *Oosp* family KO mice by genomic PCR. WT: wild-type. (**c**) Average litter size of control and triple-KO females (*p < 0.05).

To rule out any effects of compensation, we used the CRISPR/Cas9 system to excise the entire *Oosp* region. We designed two gRNAs, one in *Oosp2* and another in *Oosp3*, and purchased them as CRISPR RNA (crRNA) (Figure 3a). The two crRNA were electroporated along with the trans-activating RNA (tracrRNA) and CAS9 protein into fertilized oocytes. Ninety-three embryos were transferred into pseudopregnant female mice, and seven pups were born with a heterozygous triple KO (TKO) mutation. Subsequent mating resulted in female mice with homozygous TKO alleles (*Oosp* TKO). This deletion was verified by PCR (Figure 3b) and sequencing analysis. In the TKO mutant mice, 64,159 bp were deleted, including all of the *Oosp* family genes. *Oosp* TKO females were mated with wild-type males for several weeks. While these TKO female mice were also fertile, they had a reduced litter size (8.9 ± 2.3 in control, 6.3 ± 2.6 in TKO, *p* = 0.022) (Figure 3c).

**Figure 4:**
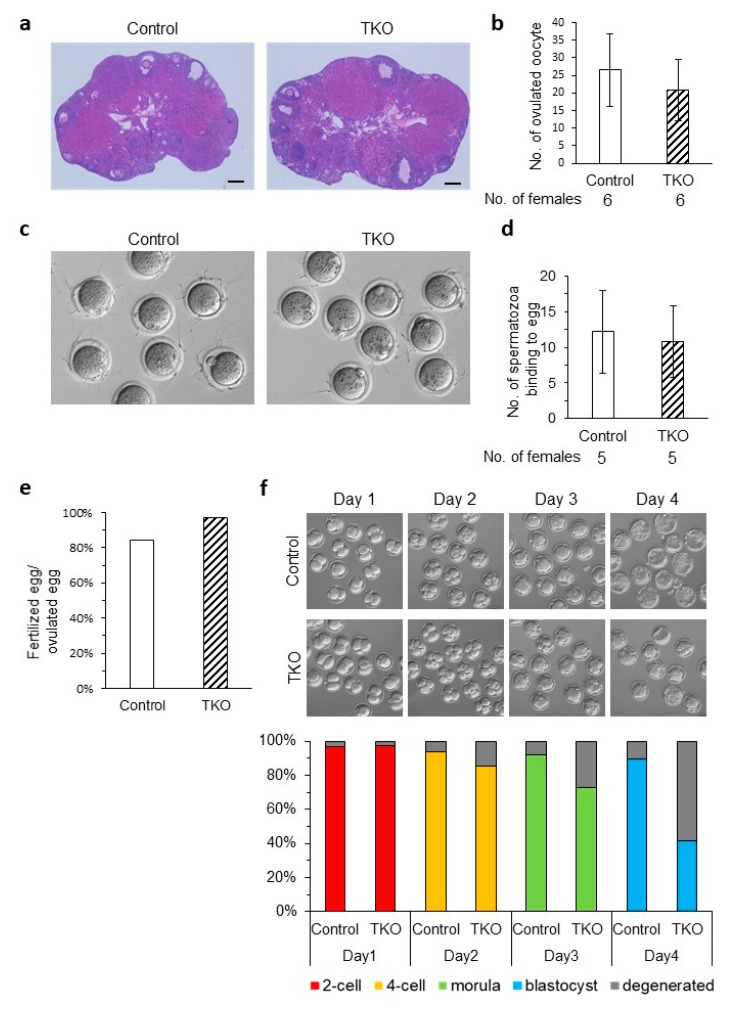
Phenotypic analysis of *Oosp* family KO females. (**a**) Histological analysis of ovaries in *Oosp* heterozygous KO (Control) and homozygous KO (TKO) mice. Scale bars = 200 µm. (**b**) Average number of oocytes ovulated by a hormone primed female. Four-week-old females were examined (*n* = 6). (**c**) Sperm ZP-binding assay. Spermatozoa were inseminated with control and KO oocytes from which cumulus were removed. (**d**) Average number of spermatozoa bound to ZP. Three-week-old to 14-week-old females were used. (*n* = 5) (**e**) Average fertilizing ratio: 133 heterozygous and 106 TKO oocytes were examined. (*n* = 5) (**f**) Developmental ratio of embryos after in vitro fertilization. Superovulated oocytes from control and TKO females were examined. Two-cell embryos, four-cell embryos, morulas, and blastocysts were observed at 24 h (Day1), 48 h (Day2), 72 h (Day3) and 96 h (Day4) after insemination. One hundred twelve and 103 eggs ovulated by four-week-old to 10-week-old heterozygous and TKO females were examined, respectively (*n* = 5, *p* < 0.05).

To understand the reason why litter size was reduced, we did a set of in vitro experiments. First, we conducted histological studies of TKO and wild-type ovaries to look at follicle development and found no significant differences (Figure 4a). Next, we wanted to see how many oocytes were ovulated by hormone-primed TKO females and found that the number was comparable to the control (20.8 ± 8.8 and 26.5 ± 10.2, respectively) (Figure 4b). Since the *Oosp* family genes all have a zona-pellucida-like domain, we wanted to see if there were any issues with ZP structure and function. Wild-type spermatozoa from the caudal epididymis of adult males were cultured in TYH medium and then mixed with MII stage cumulus-free oocytes. The eggs were then fixed and observed under the microscope. ZP structure looked normal in TKO mice, and we found that the number of spermatozoa bound to the KO oocytes was comparable to the control (12.2 ± 5.8 vs. 10.8 ± 5.0, respectively) (Figure 4c,d). We then conducted IVF to see if there were any fertilization and/or developmental defects. TKO oocytes could be fertilized by wild-type spermatozoa, and thus fertilized eggs had little variance up until the morula stage (Figure 4e,f). There is a significant drop in viable embryos created from *Oosp* TKO oocytes compared to the control between the morula stage (69% ± 21% vs. 88% ± 15%, respectively, *p* = 0.178) and the blastocyst stage (37% ± 28% vs. 86% ± 13%, respectively, *p* = 0.012) (Figure 4f). This drop correlates with a reduction in litter size.

### 3.6. Generation and Phenotypic Analysis of Cd160 and Egfl6 Mutant Mice Using the CRISPR/Cas9 System

In conjunction with the *Oosp* family KO, we also created *Cd160* and *Egfl6* KO mice using the CRISPR/Cas9 system (Figure S1). Similarly, we designed gRNAs, confirmed their activity in vitro, inserted the gRNA sequence into the pX330 plasmid, and microinjected the plasmid into the pronuclei of fertilized oocytes. Seventy-two *Cd160* and 75 *Egfl6* gRNA-injected two-cell embryos were transplanted into the oviducts of pseudopregnant female mice. Of the pups genotyped, one *Cd160 mutant* and one *Egfl6* mutant were obtained (Table S3). Subsequent mating resulted in a *Cd160* KO mouse with a 20 bp deletion and an Egfl6 KO mouse with a +1 insertion (Table S2). The primers and amplification conditions are summarized in Table S4.

Both KO mouse lines were phenotypically analyzed alongside their wild-type or heterozygous controls. The mutant mice were viable, with no overt abnormalities. To test the fertility of female mice, adult KO females were mated with wild-type males for several months. Both *Cd160* and *Egfl6* KO female mice were fertile, with averages of 6.7 ± 2.0 and 7.6 ± 1.7 pups per birth, respectively.

## 4. Discussion

By systematically knocking out genes that are conserved in mice and humans, we may one day fully explain the intricacies of the reproductive system. In the past, it was assumed that evolutionarily conserved genes must be important for the reproductive organ system they are expressed in, especially since they continue to persist in all organisms. Unfortunately, gene-editing technology reports the contrary [30,31,32]. Perhaps these genes had a purpose once individually, but many of the genes we study are not essential for the survival of the organism. As our lab continues to find more and more dispensable genes, we believe it is our obligation to the scientific community to report our findings so that other labs do not misuse precious time and resources to study the same genes.

None of this would be possible if it were not for recent technological advances in genome editing [28,29]. Thanks to the CRISPR/Cas9 system, as well as its application variants, we are rapidly producing KO mice and judging their fertility [30]. This study used two different approaches to create KO mice—intracytoplasmic injection of a CRISPR/Cas9 plasmid [16] and the electroporation method [17]. Due to transcriptional splicing variations, the genome may completely bypass the indel during transcription, leading to a fully functional protein. To address this major concern, we have now begun to delete the entire gene loci using electroporation to remove any doubt [33].

In 2001, *Oosp1* was found via cDNA hybridization arrays, and database searches showed that it shared almost 30% identity with the placenta-specific protein 1 (PLAC1) [18]. Further analysis showed that the gene was expressed specifically in oocytes in primary through antral-stage follicles. In 2005, Paillisson et al. found two additional *Oosp* family genes, *Oosp2* and *Oosp3*, with partial N-terminal zona-pellucida-like domains [19]. It was suggested that these three genes encode for structural proteins, perhaps related to the formation of the zona pellucida. After analyzing the *Oosp* family KO females, we concluded that while the fertility of the mice decreased, OOSP family proteins are dispensable for ZP structure and function. We also believe that because *Plac1* shares only 30% of its identity to the *Oosp* family and is rarely expressed in oocytes, it probably does not compensate. The *Oosp* family may play an important role for postfertilization development, which is why we saw a significant decrease in TKO prolificacy. In vitro conditions may be harsher to the developing embryo than in vivo, which could explain why the defect is more severe. It is unusual to see developmental defects in the morula–blastocyst stages in maternal gene KO mice. It is known that maternal mRNA and proteins are extremely important for early embryo development. Perhaps the OOSP family proteins, while not essential, contribute to the preimplantation development of the embryo in mice. Secreted *Oosp* proteins may function as an autocrine/paracrine factor, but further studies are required to understand their role at a molecular level.

CD160 is a protein associated with natural killer (KT) cells [34] but is also implicated in the female reproductive system. According to its FPKM profile, the CD160 protein is expressed in the oocyte. There are only a few studies on the function of CD160 in the female reproductive tract, such as one which explores CD160′s role in murine pregnancies [35]. However, the *Cd160* KO mice were fertile, indicating that *Cd160* may only play a role in immunological situations. *Egfl6* is a gene shown to be expressed in developing embryos. Published reports of this gene show that it regulates ovarian cancer [36]. Compared to normal ovaries, EGFL6 mRNA was significantly elevated in ovarian cancer histology and induced ovarian cancer cell proliferation. Unfortunately, based on our KO results, this gene does not regulate fertility.

The NOD-like receptor family is a group of genes well known for their role in immunity but has been found to have a critical role in female fertility. Many genes in the family have expression patterns in the oocyte and preimplantation embryos [37]. To highlight a few, *Nlrp2* KO oocytes had decreased developmental potential, and the gene was found to be part of the subcortical maternal complex (SCMC) [38]. *Nlrp4f* is a gene also associated with the SCMC, and KO mice had decreased fecundity as well as delayed preimplantation development [39]. Finally, *Nlrp5* KO oocytes were found to be arrested at the two-cell stage, thus essential for embryonic development [40]. Due to the power of the CRISPR/Cas9 system, we have the ability to efficiently and accurately delete family cluster genes. While the *Oosp* family was not found to be critical for fertility, we have identified multiple male reproductive tract specific family cluster proteins that are [41]. The PATE and CST families are crucial for male fertility [41]. Interestingly, a few of these genes were not essential individually but rendered the male mice to be infertile when knocked out together. Perhaps these genes become more important with the increase of copy numbers. Changes in gene dosage may play a significant role in phenotypic consequences and therefore may have evolutionary advantages.

These studies show that even though genes are evolutionarily conserved and have an in silico expression pattern linked heavily to the female reproductive tract, genes may not individually have a key role in fertility. After creating KO mouse lines using the CRISPR/Cas9 system, we found that all females demonstrated fairly normal fecundity, and thus these five genes, *Oosp1*, *Oosp2*, *Oosp3*, *Cd160*, and *Egfl6*, are individually dispensable for female fertility. However, we cannot eliminate the possibility that they may be involved in other pathways that have yet to be examined and could potentially play a more significant role in humans.

## Supplementary Materials

The following are available online at https://www.mdpi.com/2073-4409/9/4/821/s1, Figure S1: Conservation of *Cd160* and *Egfl6* between species, tissue expression analysis, and KO mice fertility, Table S1: Primers used for RT-PCR analysis, Table S2: sgRNAs used for generating knockout mice, Table S3: Efficiency of generating KO mice, Table S4: Primers used for genotyping analysis and conditions of PCR.
