# Supplementary material for: CRISPR/Cas9-Mediated Genome Editing Reveals Oosp Family Genes are Dispensable for Female Fertility in Mice
Source: Cells. 2020 Mar 28;9(4):821. doi: 10.3390/cells9040821 (PMC7226750; doi:10.3390/cells9040821)
Supplement: Supplementary file 1 [file cells-09-00821-s001.pdf]

Supplementary Material

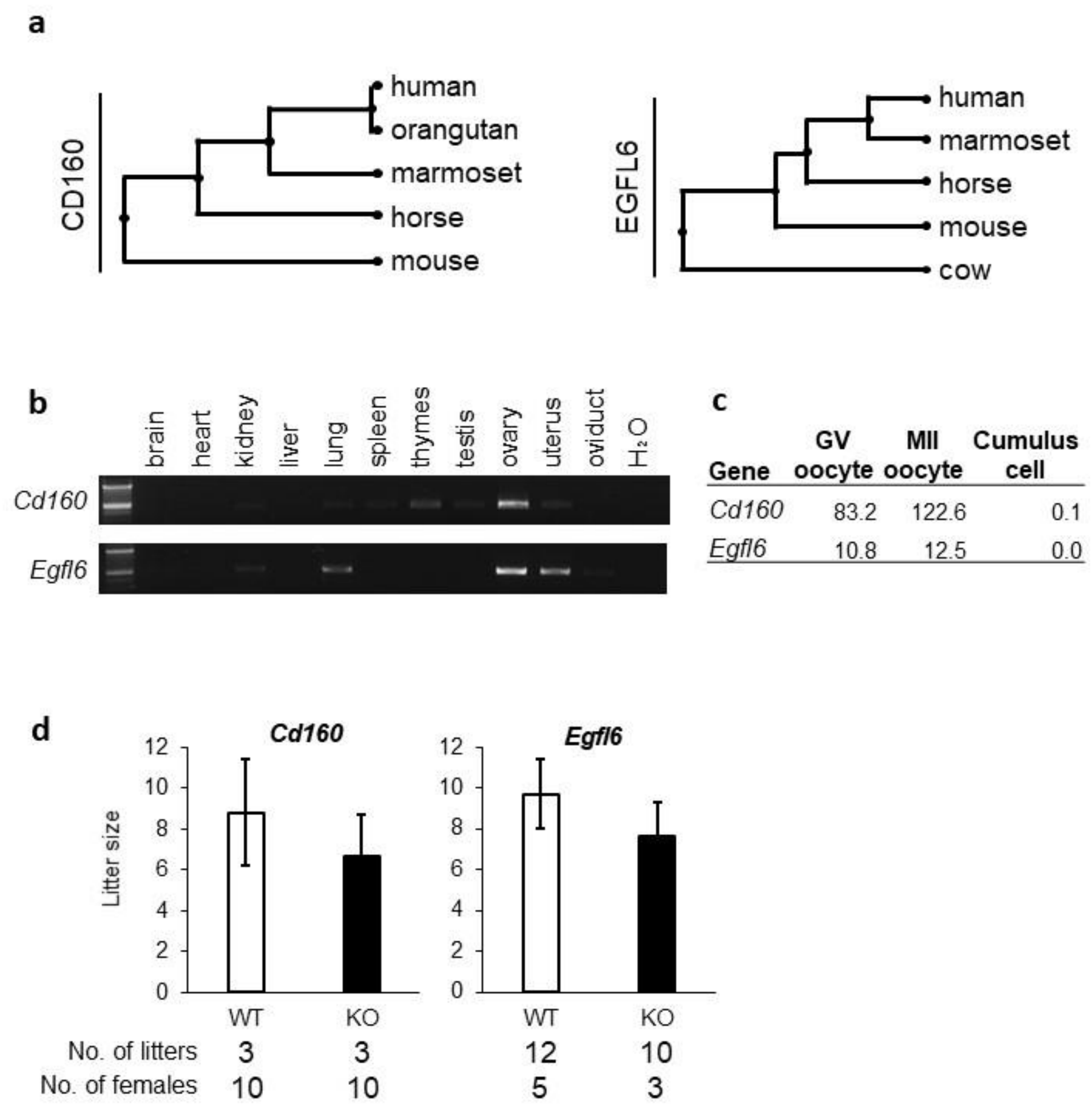

Figure S1. Conservation of *Cd160* and *Egfl6* between species, tissue expression analysis, and KO mice fertility. (a) Phylogenetic trees. (b) RT-PCR analysis using multiple tissues. (c) Expression levels in germinal vesicle (GV) oocytes, metaphase II (MII) oocytes and cumulus cells. (d) Average litter size of control and KO females.

Table S1. Primers used for RT-PCR analysis.

Gene	Upstream	Downstream	Band size (bp)
Oosp1	GTCTGCAATGTGCCGACCAC	CTCAGACCTGCTCGTTTCAG	464
Oosp2	AGTCTGCCGGATGGGGGTCT	TCCGAGAGGCAGAACACTGCAA	374
Oosp3	ATGAAGGCCTTCGTTGCTTC	AGTACCAGGAACCAGCATTG	470
Cd160	GCTGGCAATTGTGAACTTCC	AACTGAGAGTGCCGTTGATA	428
Egfl6	GGTGCAAGTTCGGTGAGTGT	CGGGTATTGAGGCAATTGGC	517
Actb	CATCCGTAAAGACCTCTATGCCAAC	ATGGAGCCACCGATCCACA	171

Table S2. sgRNAs used for generating knockout mice. sgRNAs used for generating *Oosp* family knockout mice targeted the gene's upstream (U) and downstream (D) regions. EP: electroporation

Gene	sgRNA (5'→3')		PAM	Mutation
Oosp1	–		–	COMP tm1b
Oosp2	GTGCGTGTATTTGAGTTACA		CGG	-11/-11
Oosp3	GCTGCCCAGTAACTTCCATA		AGG	-26/-26
Oosp family	U	TATGCCACTATCTACTGAGG	AGG	-64159/-64159
	D	ATGCTGCTGTGGCAAGTCAA	CGG	
Cd160	AGGGCACAGCAGCTTTGGCC		AGG	-20/-20
Egfl6	CAGCCTGGGGTCTGTCAGTA		TGG	+1/+1

Table S3. Efficiency of generating knockout mice. EP: electroporation.

**Gene**	**method**	**No. of transferred egg**	**GMO / pups genotyped**
Oosp1	COMP tm1b	*ES	*ES 24/28
Oosp2	Plasmid injection	87	2/19 (11%)
Oosp3	Plasmid injection	102	3/26 (12%)
Oosp family	EP	93	7/33 (21%)
Cd160	Plasmid injection	72	1/3 (33%)
Egfl6	Plasmid injection	75	1/6 (17%)

Table S4. Primers used for genotyping analysis and conditions of PCR.

Gene	Allele	Forward primer	Reverse primer	Annealing	Elongation	Band Size (bp)
Oosp1	WT	GTGCCGACCACTGGTTCCATC	GCACTGTTACAGCACAGCCTCTC	65℃,30s	72℃,30s	532
	KO	CACACCTCCCCCTGAACCTGAAAC	GCACTGTTACAGCACAGCCTCTC	65℃,30s	72℃,30s	331
Oosp2	WT	GTCCATCCTTTCGTCACAGCTCC	CATGCATATGGATCAGTTAAAGGACC	60℃,30s	72℃,30s	406
	KO	GTCCATCCTTTCGTCACAGCTCC	CATGCATATGGATCAGTTAAAGGACC	60℃,30s	72℃,30s	395
Oosp3	WT	AACTTCTGAGTGTTTTG	ATTATCCTATACATGGCAAG	60℃,30s	72℃,30s	577
	KO	AACTTCTGAGTGTTTTG	ATTATCCTATACATGGCAAG	60℃,30s	72℃,30s	551
Oosp family	WT	TGCCTACCTCCTGTCCTAACAACCTG	CTTGCAGTGATTCTGTTGTCTCTG	60℃,30s	72℃,30s	746
	KO	TGCCTACCTCCTGTCCTAACAACCTG	CACTGATAGTTATTTTCATAGGTATTACAAAAGG	60℃,30s	72℃,30s	366
Cd160	WT	AACACGGGCTATTCACCAAAGGAGC	GACTTTGTGAAATGCCTTGGCTC	60℃,30s	72℃,30s	653
	KO	AACACGGGCTATTCACCAAAGGAGC	GACTTTGTGAAATGCCTTGGCTC	60℃,30s	72℃,30s	633
Egfl6	WT	CTAACTCAGAATTCTTTATCCACAGTCCAT	GTGAAGTGTCAACCACTCTGGACACTGCAC	60℃,30s	72℃,30s	304
	KO	CTAACTCAGAATTCTTTATCCACAGTCCAT	GTGAAGTGTCAACCACTCTGGACACTGCAC	60℃,30s	72℃,30s	305
